# G-Force PD: a global initiative in coordinating stem cell-based dopamine treatments for Parkinson’s disease

**DOI:** 10.1038/npjparkd.2015.17

**Published:** 2015-09-24

**Authors:** Roger A Barker, Lorenz Studer, Elena Cattaneo, Jun Takahashi, G-Force PD consortium

**Affiliations:** 1 John van Geest Centre for Brain Repair, Forvie site, University of Cambridge, Cambridge, UK; 2 Memorial Sloan Kettering Cancer Center, NY, USA; 3 Department of Biosciences, University of Milan, and INGM, National Institute of Molecular Genetics 'Romeo and Enrica Invernizzi', Milano, Italy; 4 Department of Clinical Application, Center for iPS Cell Research and Application, Kyoto University, Sakyo-ku, Kyoto, Japan

## Abstract

Translating new cell-based therapies to the clinic for patients with neurodegenerative disorders is complex. It involves pre-clinical testing of the cellular product and discussions with several regulatory agencies, as well as ethical debates. In an attempt to support efforts around the world, we set up a global consortium that brings together the major funded teams working on developing a stem cell-derived neural transplantation therapy for Parkinson’s disease (PD). This consortium, G-Force PD, involves teams from Europe, USA, and Japan, and has already met on two occasions to discuss common problems, solutions, and the roadmap to the clinic. In this short review, we lay out the brief history and rationale for this initiative and discuss some of the issues that arose in our most recent meeting (May 2015) as we consider undertaking first-in-human clinical trials with stem cell-derived neurons for PD.

## Introduction

There are no curative therapies for chronic neurodegenerative conditions. These conditions include Alzheimer’s disease, Parkinson’s disease (PD), Huntington’s disease (HD), FrontoTemporal Dementia, and amyotrophic lateral sclerosis. Of these, PD is the most amenable to therapy given that part of its pathology is defined by the loss of nigral dopaminergic neurons, which means that dopaminergic therapies can be employed with great success at least in the early stages of the condition.^1^ Pharmacological dopamine replacement therapy, which began over 50 years ago, shows that dopamine replacement is transformative, albeit not curative, for PD patients. Furthermore, when given in the form of oral therapies, it leads to non-physiological stimulation of dopamine receptors. This in turn leads to the common, and potentially severe, side effects including L-DOPA-induced dyskinesias (LIDs), as well as psychiatric and cognitive problems. Such complications have been addressed by many new therapies including deep brain stimulation (DBS) and continuous delivery of dopaminergic agents (e.g., Apomorphine and DuoDopa infusions).^2^

Despite these therapeutic triumphs, the pathogenic process underlying PD continues. After 10–15 years, most patients exhibit numerous motor complications and a range of non-motor deficits. Consequently, there is a desperate need for effective disease-modifying therapies, but until such a therapy is available; an alternative approach is to try to repair the dopaminergic nigrostriatal system. One such approach to brain repair involves grafting dopaminergic neurons to replace those lost to the original disease process.^3^

Neural grafting for PD has a long history going back to the late 1970s, when it was shown that immature dopamine neurons could restore dopamine levels and mitigate behavioral deficits in animal models of PD. Dopaminergic neurons derived from the fetal ventral mesencephalon (fVM) of the appropriate developmental age (embryonic day 13–14) survived grafting in the adult rodent striatum, extended axons into the transplanted brain, and formed synapses with host neurons. In addition the grafts restored tissue levels and release of dopamine in the striatum, which was coupled to normalization of drug-induced and spontaneous behavioral deficits.^3^ This was reproduced in many laboratories worldwide and in 1987 led to the first clinical trials in patients that ran for about another 13 years.^4^ In these studies, patients typically received intrastriatal grafts of human fVMs derived from 1 to 8 aborted foetuses (aged 7–9 weeks post conception) implanted into each side of the brain. The patients were often in moderate to advanced stages of PD with already manifest LIDs.

The original clinical studies were open label and showed that some patients in receipt of such transplants did well for many years and could even reduce or discontinue their anti-PD medication. In the most successful cases the striatal F-DOPA uptake, monitored by positron emission tomography (PET), returned to normal levels 3–5 years after surgery.^5^ However, not all patients improved and the optimal patient population and delivery approach using this fetal tissue was still not fully resolved when two double-blind placebo controlled studies were undertaken in the USA, supported by the National Institutes of Health. These clinical studies, in contrast to the open label studies, found no significant benefit from the grafts. In addition, many of the patients developed a complication from the procedure, namely graft-induced dyskinesias.^6,7^ The publication of these results at the beginning of this century led to a moratorium on this approach, especially as newer more easily applied therapies such as DBS became available.

In order to try to better understand these disparate findings, an international working group was set up in 2006 supported by Parkinson’s UK, to which all those involved in such trials were invited. These meetings over the ensuing 2 years sought to analyze the data to see whether lessons could be learnt from the trials that had already taken place and then to use those data to inform the future development of these therapies in the clinic. This collaborative approach enabled the community to agree that the patients who derived most benefit from these grafts were generally younger, earlier in their disease course and in receipt of transplants derived from at least three human fVMs. This information was then used to design a new trial looking at this approach in PD, TRANSEURO, which was funded by the European Union (EU) in 2009 and led to the first transplants being performed in 2015.

This whole process taught us two main things: (i) working as a collaborative group enabled conflicting views to be discussed and debated, which ultimately improved the rationale and approach adopted in taking such therapies forward; and (ii) the regulatory landscape when using such cells is complex and often ill-defined, and working together as a group with the relevant authorities sped up and informed the whole process. This collaborative approach with regards to fetal grafting led to the idea that those working with multiple types of stem cell-derived dopaminergic cell therapies for PD may also benefit from working together as a consortium.^8^

## G-Force PD—ITS origins

**Figure 1:**
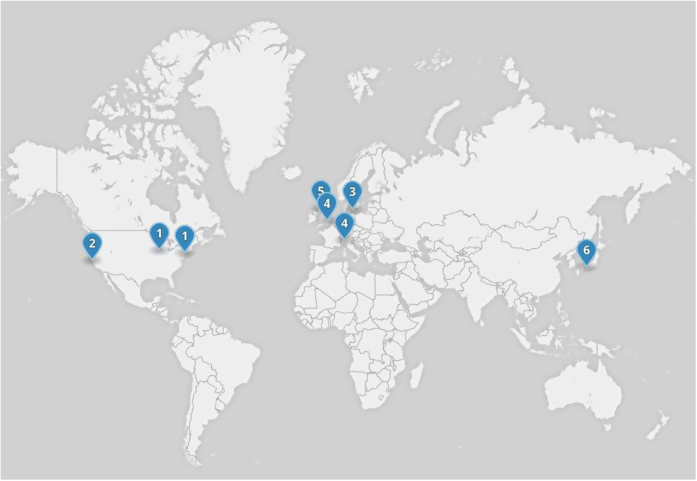
The geographical distribution of the relevant parties involved in G-Force PD. 1=NYSTEM consortium involving centers in New York and Chicago; 2=CIRM; 3=Lund University who are part of TRANSEURO and NEUROSTEMCELLREPAIR; 4=University of Milan that co-ordinates the NEUROSTEMCELLREPAIR consortium; 5=A number of UK sites including the Universities of Cambridge, Cardiff, and London who are involved in NEUROSTEMCELLREPAIR and TRANSEURO; 6=CiRA.

The teams that originally sought to work together to develop this new consortium were drawn from those groups who were in receipt of significant funding for such programs of work (see below and Figure 1).

- TRANSEURO—a Framework Program 7 (FP7) EU funded consortium that seeks to reintroduce fetal VM allografts into clinical trials with patients with PD (http://www.transeuro.org.uk/);
- NEUROSTEMCELLREPAIR (NSCR)—an FP7 EU funded consortium that seeks, as one of its main goals, to develop good manufacturing practice (GMP) grade dopaminergic neurons from stem cell sources for eventual use in patients with PD (http://www.neurostemcellrepair.org/; http://www.crm.ed.ac.uk/research/group/mechanisms-neurodegeneration);
- NYSTEM have awarded a contract to L.S., Viviane Tabar and colleagues at Memorial Sloan Kettering (MSKCC) in New York to develop a human embryonic stem cell-derived clinical grade dopaminergic midbrain neuron for use in clinical trials in PD (https://www.mskcc.org/research-areas/programs-centers/new-york-state-stem-cell-science-consortia);
- CiRA—a Japanese research centre that is funded to do many types of regenerative cell-based therapies. In particular, Jun Takahashi is funded to develop a clinical grade dopaminergic cell therapy from autologous-induced pluripotent stem cells (iPS cells) taken from the PD patients (https://www.cira.kyoto-u.ac.jp/e/);
- The California Institute for Regenerative Medicine (CIRM), given their involvement in funding regenerative cell-based therapies in California, even though they are not currently funding a major ongoing clinical PD programs in this area.

Other organizations/groups that also came to the meetings included:

- Other parties with a vested interest in this field including, e.g., representatives of the two major US PD-related charities—the Michael J. Fox Foundation for Parkinson's Research (MJFF) and the Parkinson's Disease Foundation (PDF);
- A Medical Research Council (MRC) funded project led by Tilo Kunath in the UK that seeks to apply established dopaminergic differentiation protocols to existing UK derived human GMP grade Embryonic Stem (ES) cell lines.

The first of these meetings took place in London in May 2014 with a second meeting in New York in 2015. Both meetings lasted 1.5 days and involved each group presenting their own program of work and how they were aiming to take this therapy to clinic. These presentations were followed by discussions on the problems and challenges that lay ahead in taking this therapy to patients and a roadmap to the first clinical trial (http://www.gforce-pd.com/). A similar meeting is in the planning stages to take place in Chicago in 2016. The funding for each meeting was met from funds within their consortia, as well as support from the Parkinson’s UK in the first meeting in London and the PDF and Weill Cornell for the meeting in New York.

## G-Force PD—the challenges

The main challenges that were addressed at each meeting were:

- The pre-clinical data defining all the requirements of a stem cell-derived dopaminergic neuron making it sufficiently likely to be efficacious and safe so it can be considered for use in a clinical trial. This has been the subject of a number of recent reviews.^9,10^ In essence, before any stem cell-derived dopaminergic neuron can be considered suitable for patients with PD, it needs to express a number of basic characteristics. This not only embraces issues around the stem cell source (e.g., which ES cell line, genetic stability, minimal or no risk of zoonoses) but also the identity and stability of the dopaminergic neurons derived from the stem cell line. To date, there is no clear regulatory path in either Europe or in the USA as those that exist were created before pluripotent stem cell therapies were developed. Despite this ambiguous regulatory landscape, a number of groups have entered clinical trials using pluripotent stem cell-derived products in USA, UK, and Japan. In Europe, it is preferred that the starting material (i.e., ES cell line) is created using the highest standard of material available, specifically that the original line be derived under GMP conditions, with appropriate consent and processed throughout in xeno-free conditions. However, there are some trials already underway in which the starting materials do not reach these standards. In USA, a number of trials have been initiated in which the starting material is considered ‘research grade’ and a xenogeneic product. Of greater concern in USA is the lack of adequate testing for transmissible spongiform encephalopathies—rendering all cell lines derived from UK donors non-compliant with US Food and Drug Administration (FDA) donor eligibility requirements (Title 21 Code of Federal Regulations, part 1271). As such, it is likely that the ES cell lines initially used for clinical trials in USA and Europe may be different, while in Japan the focus is on using patients' own cells to make autologous iPS cell lines. At the time of writing, though, there appears to be a shift in emphasis away from patient specific to previously banked Human Leukocyte Antigen-typed iPS cell lines in this country, due to complications in patient-by-patient banking.
- The cell production processes in the pre-clinical work can now yield differentiated cells that express an array of relevant markers for midbrain dopaminergic neurons with fiber outgrowth.^11,12^ However, it is still debated what markers these cells should express and at what levels. Nevertheless, in all cases the dopaminergic neurons produced by the G-Force PD partners appear to display the critical morphological, neurochemical, and transcriptional markers of appropriate cells, with pacemaker potentials, dopamine release, and functional integration and effects in animal models of PD without tumor formation or overgrowth. However, in all cases questions still arise as to whether the manufacturing and differentiation protocols for generating these cells have been optimized in each center (or at least developed to a level to support a clinical trial). This is critical because it is only at this point that the process can be locked down and moved to GMP.
- The necessary GMP manufacturing processes to take the cell to a clinical trial. Entry into a phase 1 trial does not necessarily require all reagents to be of GMP grade, but they must be properly sourced and tested. This is often thought to be relatively straightforward, but represents a major hurdle as the relevant reagents are in some cases extremely hard, if not impossible, to find. If they can be sourced, the cost is often much greater than equivalent research grade material. Furthermore, the change from a research grade reagent to one of an equivalent GMP grade can affect the whole differentiation outcome. Thus, many experiments, including validations *in vivo*, need to be done to verify that the original lab-based protocols still work under GMP conditions. All groups are now tackling this issue and sharing reagents where possible, also in part to disperse cost. Assay development (for quality and batch control) is also critical in demonstrating a robust and reproducible process and product. Like the conversion to GMP compliant protocols, it is often underestimated and requires extensive effort and cost.
- Issues concerning cell sorting and cryopreservation of the final product. Cell sorting has the advantage that it can select out cells that are not wanted (non-dopaminergic neurons or neural progenitors) and/or positively select for the wanted cells. All of this runs the risk that many cells are lost in the process and having a pure dopaminergic neuronal population as a final cell product, without some supportive glial cells, may not be ideal. Cell sorting also introduces an additional complication into any manufacturing process. In the case of using positive sorting, one must consider the quality of the antibody used, as it may be carried along with the cell product, at least transiently. GMP grade antibodies are also expensive to make and take a considerable time to be generated. Cryopreservation of cells seems to require dimethyl sulfoxide (DMSO), and while low levels can be tolerated in cells delivered peripherally to patients for other medical reasons, there is less information about how well residual levels of DMSO may be tolerated following injection of the cells into the brain.
- Regulatory processes required in taking any new stem cell-based trial into the clinic for PD. There are many unknowns in taking a new therapy to clinic that are almost impossible to answer in the laboratory, and these include the long term survival of the cells (over years) and their stability, as well as their immunogenicity in the human brain. Thus, early discussions with the relevant regulatory authorities have already begun for many in the consortium, to see what safety studies will be required, including the need for cell sorting (see above), as well as non-human primate grafting experiments. This may vary between countries with the FDA differing from the European Medicine Agency and Japanese authorities in what they would want to see in the final product submission for a clinical trial.
- The structure of a Phase I clinical trial using this type of cell product. As this will be a first-in-human study, a balance will need to be struck between grafting patients with late stage disease, who may have exhausted other options, against those most likely to benefit from the therapy, who may be younger and have less severe symptoms.^13^ Although an argument can be made that the ideal patient population was defined in the ongoing TRANSEURO study with human fVM tissue, it is more likely that the upcoming phase 1 studies will involve patients with relatively advanced disease who experience motor fluctuations, but nonetheless remain free of major non-motor problems. This was the case for the patients selected for the initial human fVM grafts and clearly gave early indications on the magnitude of effects that such grafts could exert. These first trials will focus on tolerability and feasibility endpoints, including magnetic resonance imaging screening to monitor for graft overgrowth. In addition, signs of efficacy will clearly be examined both clinically and through imaging using PET ligands for the dopaminergic system. The exact clinical trial designs for the US and the European groups are currently being discussed, but may be modified depending on what the regulatory authorities' request, and this extends to the dose of cells that will be grafted. In the Japanese studies, the patients will need to be identified early on if the decision is made to use autologous iPS cells as the source of the dopaminergic neurons for grafting.^14^
- Developing bioethical underpinnings for a first-in-human cell transplant trial. Trials of this nature present a variety of ethical challenges that require careful attention. Among such challenges are decisions about: how much and what type of evidence is required before proceeding to experimentation in human beings; how to ensure that risks to participants are minimized while potential benefits to them and society are maximized; how to design an informed consent process that enables participants’ to make truly autonomous decisions; and how to limit and manage conflicts of interests. These ethical challenges are not unique to stem cell transplantation trials. However, the publicity that surrounds such research, the great promise that these potential treatments present, the significant uncertainty about risks and potential benefits, and the devastating consequences that an initial failed trial could have for the stem cell field, all call for particular care in addressing these ethical challenges.
- Involving the broader community. PD-related charities and foundations already have a valuable role in enhancing the role of the public in developing clinical research enterprises. In addition to providing educational resources on research efforts, some support development of patient research advocates. Such engaged individuals provide a direct link to the PD community, support advocacy efforts, and may aid in shaping aspects of clinical trials, for example developing information materials that will support trial participants from the informed consent process and beyond.
- Ability to encourage others to be a part of this or similar consortia. While this meeting sought to bring together many of the relevant groups in this field, there are other privately funded enterprises seeking to do similar work. Although it may not be possible for all to participate in such meetings at this time, given the confidential nature of their work, it is hoped that they will seek to follow similar approaches to those we are collectively adopting to help prevent a premature translation to the clinic.
- The commercialization of the cell product and its ultimate position in the clinic. While this remains some way off, it is important to recognize that academic institutions and their principal investigators can only develop these therapies so far. Larger phase 2 and 3 studies will be prohibitively expensive and will require a major investment from industry. With this will come issues of intellectual property (IP), the ownership of the cell product and a loss of control of the development of that product in terms of trial design, disease indication and marketing. Thus, although there is little one can do to change this process, securing good IP early on, and remaining active in the appropriate promotion of the cell product by those involved in its early development will be vital if these treatments are to be competitive and useful to patients.

## Conclusion

This second meeting of G-Force PD served to once more highlight the value of sharing knowledge and expertize around a common goal relating to an exciting, but unproven, experimental cell therapy for PD. It is hoped that others working in similar fields (e.g., gene therapies for neurodegenerative disorders; cell therapy for HD and so on) may also wish to adopt such an approach, and by so doing help prevent premature clinical trials, while also ensuring that the work moves forward as quickly and efficiently as possible. By doing so, it should reach its ultimate goal at a time when all those best qualified to assess it, agree that this is the case, and only at such a time.

## Members of G-Force PD

G-Force consortium consists of:

- TRANSEURO–Roger Barker, Anders Björklund, Stephen Dunnett, Patrik Brundin^#^, Malin Parmar;
- NEUROSTEMCELLREPAIR–Elena Cattaneo, Ernest Arenas, Gianni Munizza, Malin Parmar, Agnete Kirkeby, Oliver Brüstle, Andreas Bosio, Roger Barker, Stephen Dunnett;
- MSKCC/NYSTEM–Lorenz Studer, Viviane Tabar, Mark Tomishima, Isabelle Rivière, Urs Rutishauser, Abderrahman El-Maarouf, Stefan Irion, Jeffrey H. Kordower*, Dustin R. Wakeman*, Claire Henchcliffe**, Inmaculada de Melo-Martin***
- CiRA–Jun Takahashi, Asuka Morizane, Daisuke Doi, Tetsuhiro Kikuchi;
- CIRM–Catherine Priest.

Others:

- MJFF–The Michael J. Fox Foundation;
- PDF–Parkinson’s Disease Foundation;
- MRC–UK Grant; Tilo Kunath.

^#^Center for Neurodegenerative Science, Van Andel Research Institute, MI, USA.

*Rush University Medical Center, Department of Neurological Sciences. Chicago, IL 60612, USA.

**Weill Cornell Medical College, Department of Neurology, New York, NY 10021, USA.

***Weill Cornell Medical College, Division of Medical Ethics, New York, NY 10065, USA.
